# Supplementary material for: Identification of photoperiod-induced specific miRNAs in the adrenal glands of Sunite sheep (Ovis aries)
Source: Front Vet Sci. 2022 Jul 22;9:888207. doi: 10.3389/fvets.2022.888207 (PMC9354845; doi:10.3389/fvets.2022.888207)
Supplement: Supplementary file 1 [file Data_Sheet_1.ZIP › Supplementary file/miRDeep2 file.zip!/miRDeep2 file/Novel_Structure/SP42A2/Novel_308.pdf]

Provisional ID	:	NC_019460.2_9523
Score total	:	7.9
Score for star read(s)	:	3.9
Score for read counts	:	0.6
Score for mfe	:	1.8
Score for randfold	:	1.6
Score for cons. seed	:	
Total read count	:	13
Mature read count	:	9
Loop read count	:	0
Star read count	:	4

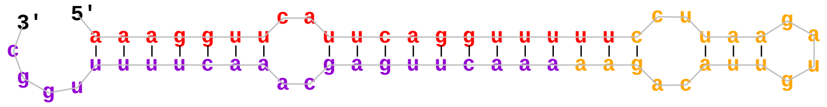

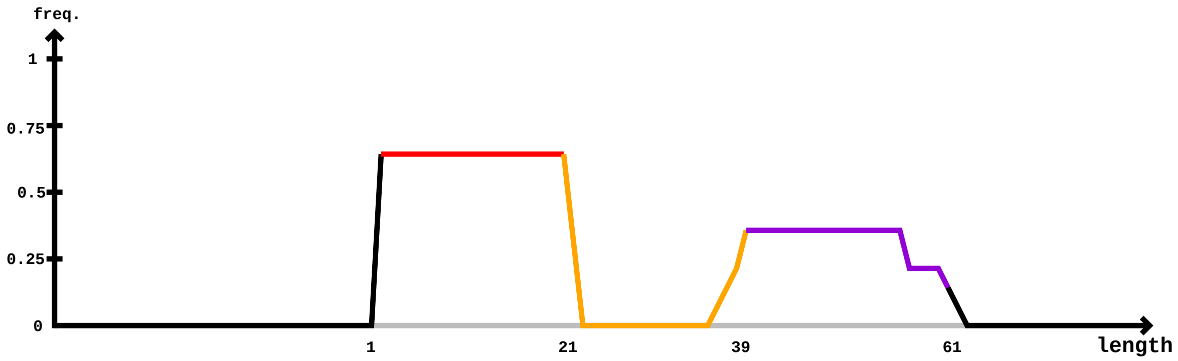

Mature **Star**

```
5'- uuuugacauuuggagaaauauuggguuacccaaaagguucauucagguuuuuccuuaagauguuacagaaaaacuugagcaaacuuuuuggccaacccaguagaguuucugc   -3'    obs
    uuuugacauuuggagaaauauuggguuacccaaaagguucauucagguuuuuccuuaagauguuacagaaaaacuugagcaaacuuuuuggccaacccaguagaguuucugc          exp
    ...........(((((..(((((((((..((((((((((..((((((((((((..(((....)))..))))))))))))..))))))))))..)))))))))...))))).. reads    mm    sample
    ................................aaagguucauucagguuuuu............................................................ 6        0     seq
    ................................aaagguucauucagguuuuC............................................................ 1        1     seq
    ................................aaagguucauucagguuuuuc........................................................... 2        0     seq
    ...................................................................gaaaaacCugagcaaacuuu......................... 1        1     seq
    ....................................................................aaaaacCugagcaaacuuu......................... 1        1     seq
    .....................................................................aaaacCugagcaaacuuuuugg..................... 1        1     seq
    ......................................................................aaacuugagcaaacuuuuuggc.................... 2        0     seq
```
